# Does Colpocleisis Still Hold Value? The Evolution of Apical Prolapse Surgery: A Comparative National Database Study †

**DOI:** 10.3390/jcm14051414

**Published:** 2025-02-20

**Authors:** Yaman Degirmenci, Ina Shehaj, Matthias Alexa, Gilbert Georg Klamminger, Mona Wanda Schmidt, Konstantin Hofmann, Annette Hasenburg, Roxana Schwab

**Affiliations:** 1 Department of Gynecology and Obstetrics, University Medical Center of the Johannes Gutenberg University, 55131 Mainz, Germany; matthias.alexa@unimedizin-mainz.de (M.A.); gilbert.klamminger@unimedizin-mainz.de (G.G.K.); mona.schmidt@unimedizin-mainz.de (M.W.S.); konstantin.hofmann@unimedizin-mainz.de (K.H.); annette.hasenburg@unimedizin-mainz.de (A.H.); roxana.schwab@unimedizin-mainz.de (R.S.); 2 Department of Obstetrics and Gynecology, University of Frankfurt, 60590 Frankfurt am Main, Germany; inashehaj@hotmail.com

**Keywords:** colpocleisis, obliterative surgery, apical pelvic organ prolapse

## Abstract

**Background/Objectives:** Pelvic organ prolapse (POP) is a common condition that significantly affects quality of life. Obliterative surgery, such as colpocleisis, represents an alternative to reconstructive surgery with a historically established background. The trends in apical prolapse surgery have undergone substantial changes following FDA warnings. This study aims to analyze the trend of obliterative surgery within the context of apical POP surgery in the German healthcare system, considering global shifts, particularly following FDA warnings. **Methods**: A comprehensive analysis of in-patient data from the German Federal Statistical Office was carried out for the period between 2005 and 2021. The study included a total of 530,107 procedures, each classified by specific codes. Linear regression analysis was applied to identify and characterize trends in surgical patterns. **Results**: The trends in obliterative surgery showed a significant decline over the years (*p* < 0.001), particularly notable in older women. In contrast, a significant increasing trend was observed in the proportion of abdominal surgeries relative to the total number of procedures over time (*p* < 0.001), especially in the younger age group. **Conclusions**: Surgical trends over the specified timeframe highlight the notable evolution of POP management. Despite observed global fluctuations, obliterative surgery in Germany appeared to follow a declining trend in the changing mesh era, shaped by varying perspectives on the matter. The shifting global trend should be closely monitored and considered in urogynecological training.

## 1. Introduction

Human beings, like all living organisms, inherently experience the process of aging. Nonetheless, when assessed by the criteria outlined in the International Classification of Diseases (ICD), aging may meet the qualifications to be classified as a disease [1]. Aging, viewed from this perspective as a significant public health concern, also serves as the primary risk factor for pelvic floor disorders in women within the field of urogynecology [2]. The recent data indicate that 25% to 50% of women are affected by one or more pelvic floor disorders (PFDs), which refer to a category of medical conditions that include pelvic organ prolapse (POP), urinary incontinence, and fecal incontinence [3,4]. Aging, alongside other leading risk factors, is strongly associated with POP, which can affect over two-thirds of women, with approximately 5% of the population experiencing symptoms [5].

Global health burden analyses highlight a significant increase in the prevalence of POP among the elderly, reflecting the shifting demographics of an aging population [2]. Forecasting models predict a further rise in the prevalence of PFDs in the coming years, driven by these demographic shifts [6]. The fastest-growing demographic segment in developed countries consists increasingly of women aged 65 years and older [7]. This aging trend is also visible globally, with an expected 19% of the global population over 80 by 2050 [8]. As the population ages, the rates of surgical interventions for POP are expected to increase, potentially leading to more recurrence interventions. From 2010 to 2050, the incidence of surgical interventions among women is projected to rise significantly, with an estimated 47.2% increase in the number of women undergoing surgery for POP [7]. Consequently, urogynecologists will face an increasing number of elderly patients requiring surgery as life expectancy continues to rise.

When surgical intervention is required for the management of POP, the primary approaches consist of reconstructive and obliterative techniques. Historically, surgeons have relied on native tissue through vaginal reconstructive procedures such as colporrhapies with diverse apical suspension techniques. All were performed both with and without hysterectomy. As an alternative, colpocleisis was introduced in the 19th century as a surgical method to treat POP by obliterating the vaginal canal. Initially performed by Neugebauer in Warsaw in 1867 and later published by LeFort in Paris in 1877, colpocleisis continues to be utilized today with only minor modifications [9].

In contrast to colpocleisis, reconstructive surgery via native tissue aims to restore the normal anatomy of the pelvic organs. However, due to high recurrence rates associated with such repairs, abdominal approaches, such as sacrocolpopexy, were introduced in the second half of the 20th century and later adapted for laparoscopic use [10,11]. In this vein, in the early 2000s, mesh repair was adopted for POP surgery via the vaginal route [12]. This approach gained popularity as a promising method to reduce recurrence risks associated with prolapse surgery. Nonetheless, in response to the rising complication rates linked to transvaginal mesh implants, the FDA issued warnings and prohibited their sale in 2019 [13]. In contrast, the Scientific Committee on Emerging and Newly Identified Health Risks (SCENIHR), the European Urology Association (EAU), and the European Urogynecological Association (EUGA) have issued positive statements supporting the use of transvaginal meshes for treating urinary incontinence and POP [14]. These varying perspectives have significantly influenced surgical approaches and treatment options for POP globally. While surgical approaches to treating female POP have evolved, the demographics of the patient population have also shifted over the past century.

Some data indicate that trends in prolapse surgery, particularly in certain countries, have shifted following the ban on mesh use. Initially declining before the 2000s, the ‘historical’ obliterative surgery, particularly colpocleisis, has interestingly regained significance and become increasingly common among older women. This trend may reflect the influence of the availability of treatments for POP following FDA notifications [15,16,17]. In our clinical practice, however, we observe that obliterative surgery, even among older patients, has become increasingly rare and is performed far less frequently than in the past. An analysis of national data from Germany covering the period of the changing mesh era revealed that, unlike in Anglo-Saxon countries, transvaginal mesh techniques have increasingly been used as a reconstructive treatment option, particularly among older patients [14].

Traditionally, colpocleisis is recommended for older women who are not sexually active, given its implications that make vaginal intercourse impossible [18,19,20]. Is colpocleisis still accurate in its current form, and can this perspective be generalized in this way despite significant changes in demographics over the past century? Recent data show that nearly one-third of individuals aged 60 to 82 report more sexual activity and thoughts than the average younger adult [21]. The rate of sexual activity remains above 30% for women aged 75 and older [19].

The study aims to investigate this question and examine surgical trends, especially obliterative procedures for apical POP in Germany. It mainly focuses on the impact of mesh controversies and demographic changes. This study hypothesizes that the trend for obliterative surgery in Germany differs from that of other countries, partly due to the continued use of transvaginal meshes, even among the aging population.

## 2. Materials and Methods

In this retrospective analysis, we utilized data from the German Federal Statistical Office, which provide detailed annual information on surgeries classified by operation (OPS) codes for inpatients without specific medical indications. To create a systematically classified dataset, we analyzed all OPS codes related to reconstructive apical prolapse surgeries of any kind and obliterative vaginal prolapse surgery, explicitly focusing on colpocleisis, over the period from 2005 to 2021. Subgroups, including colpectomies and other nonspecifically defined procedures under the primary OPS code 5-703—covering vaginal closure procedures as well as subtotal or total colpectomies—were excluded from consideration. Although OPS codes are revised annually, the coding for colpocleisis remained consistent and unchanged throughout the study period from 2005 to 2021.

After determining the total number of colpocleisis cases, we calculated and included the total number of abdominal and vaginal apical prolapse surgeries. This calculation is performed to identify trends associated with changes in colpocleisis cases and to facilitate a more accurate comparison. Since OPS codes have been updated and refined over the years covered by this study, we considered all abdominal prolapse procedures, irrespective of whether they were performed with or without mesh and utilizing either laparoscopic or abdominal techniques (Table 1). This approach was necessary because, in earlier years, coding practices were less specific, and procedures were often grouped under a general primary code. In contrast, coding practices evolved by 2021 to become more detailed and specific. To estimate the total number of cases, we subsequently included those involving vaginal prolapse treatments, specifically vaginal apical prolapse surgeries performed with or without mesh, to allow for a comparison of trends. As the database does not provide specific indications, hysterectomies that may have been performed for prolapse treatment were excluded. However, Douglas reconstruction, a common component of vaginal hysterectomies, was included in the study data as an indicator of apical prolapse surgery. Independent of the total case numbers, proportions and relative ratios were calculated to represent trends over time graphically.

To assess how age distribution impacts the number of surgical procedures performed, the data were categorized into five distinct age groups: individuals under 50 years (<50), those aged 50–60 years (50–60), 60–70 years (60–70), 70–80 years (70–80), and those over 80 years (>80). Univariate linear regression analyses were conducted with time as the independent variable, using SPSS Version 25 to identify trends within the data. A *p*-value of less than 0.05 was considered statistically significant.

A literature review was conducted to compile accessible comparative data through systematic PubMed/Medline database searches. Subsequently, ratios were used to evaluate and determine trends and compare these surgical approaches with other national surgical trends. This included a comparative evaluation of trends and was primarily based on narrative commentary. In compliance with German laws and regulations, ethics approval was not required as only aggregated and anonymized data were used. A GenAI—ChatGPT 3.5 (OpenAI; San Francisco, CA, USA)—tool was used to grammar-check the paper and improve its readability.

## 3. Results

A comprehensive analysis was conducted on a dataset of 530,107 surgeries identified by their corresponding OPS codes, covering the period from 2005 to 2021. The majority of apical prolapse repairs were performed via the vaginal route as reconstructive surgery, totaling 377,345 procedures, which accounts for 71.1% of all surgeries. The second most common approach was the abdominal route as reconstructive surgery, with 139,485 cases representing 26.3% of the total surgeries. Colpocleisis counts as an obliterative procedure comprised only a small portion, with 13,277 cases recorded, corresponding to 2.5%.

The analysis of vaginal surgeries also included enterocele repairs and Douglas reconstruction, performed as culdoplasty. Douglas reconstruction, identified by a specific OPS code since 2005, accounted for one-fifth of all vaginal surgeries during the study period from 2005 to 2021 (n = 61,431), making up 16.8% of the total number of vaginal procedures. However, the percentage of Douglas reconstruction procedures decreased over time, dropping from 25.7% in 2005 to 14.7% in 2021. Similarly, a total of 50,583 enterocele repairs were performed using the vaginal route, representing 13.4% of all vaginal surgeries in the same timeframe. Analyzing these procedures in the context of vaginal surgeries alone, enterocele repairs also showed a declining trend from 2005 to 2021.

**Figure 1:**
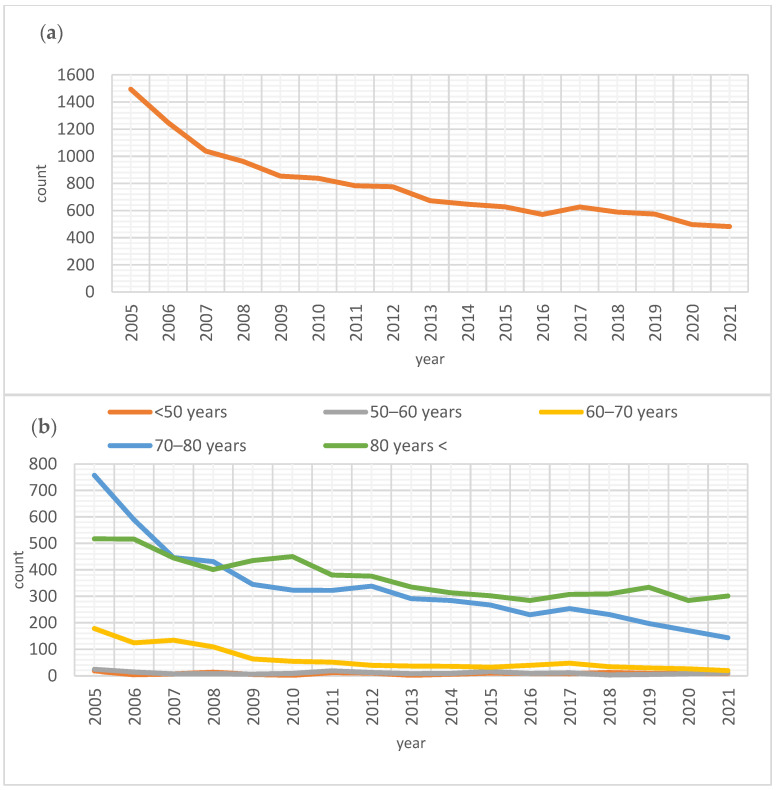
Surgical trends for colpocleisis. (**a**) Total number of colpocleisis procedures over the years (β = −0.909, *p* < 0.001, R^2^ = 0.82). (**b**) Total number of colpocleisis procedures over the years among various age categories: >80 years (β = −0.903, *p* < 0.001, R^2^ = 0.803), 70–80 years (β = −0.890, *p* < 0.001, R^2^ = 0.777), 60–70 years (β = −0.843, *p* < 0.001, R^2^ = 0.690), 50–60 years (β = −0.426, *p* = 0.088, R^2^ = 0.127) and <50 years (β = −0.015, *p* = 0.95, R^2^ = −0.066).

In the context of obliterative vaginal surgery, particularly colpocleisis, there has been a significant decrease in the total number of surgeries performed from 2005 to 2021 (β = −0.909, *p* < 0.001, R^2^ = 0.82). This decline indicated a noteworthy trend toward fewer surgical interventions during the study period. The decrease in surgeries was prominent among all age groups aged 60 and above. The decline was substantial for those aged 80 and older (β = −0.903, *p* < 0.001, R^2^ = 0.803). Similarly, individuals between 70 and 80 years showed a decrease (β = −0.890, *p* < 0.001, R^2^ = 0.777), as did those aged 60 to 70 (β = −0.843, *p* < 0.001, R^2^ = 0.690) (Figure 1).

In contrast, the number of surgeries in younger age groups experienced a less pronounced decline, mainly due to the already low rates of these procedures. Specifically, in the 50 to 60 age group, only a modest reduction was noted (β = −0.426, *p* = 0.088, R^2^ = 0.127), while for those under 50, the change was negligible (β = −0.015, *p* = 0.95, R^2^ = −0.066) (Figure 1).

**Figure 2:**
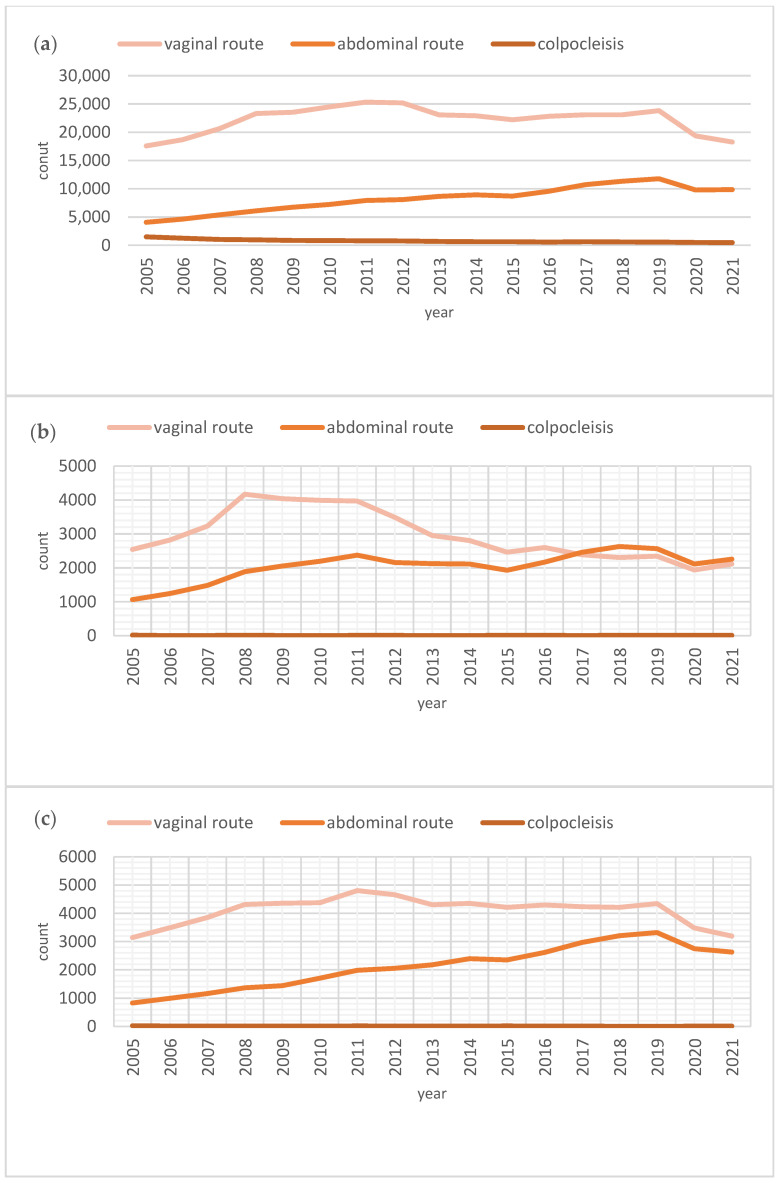

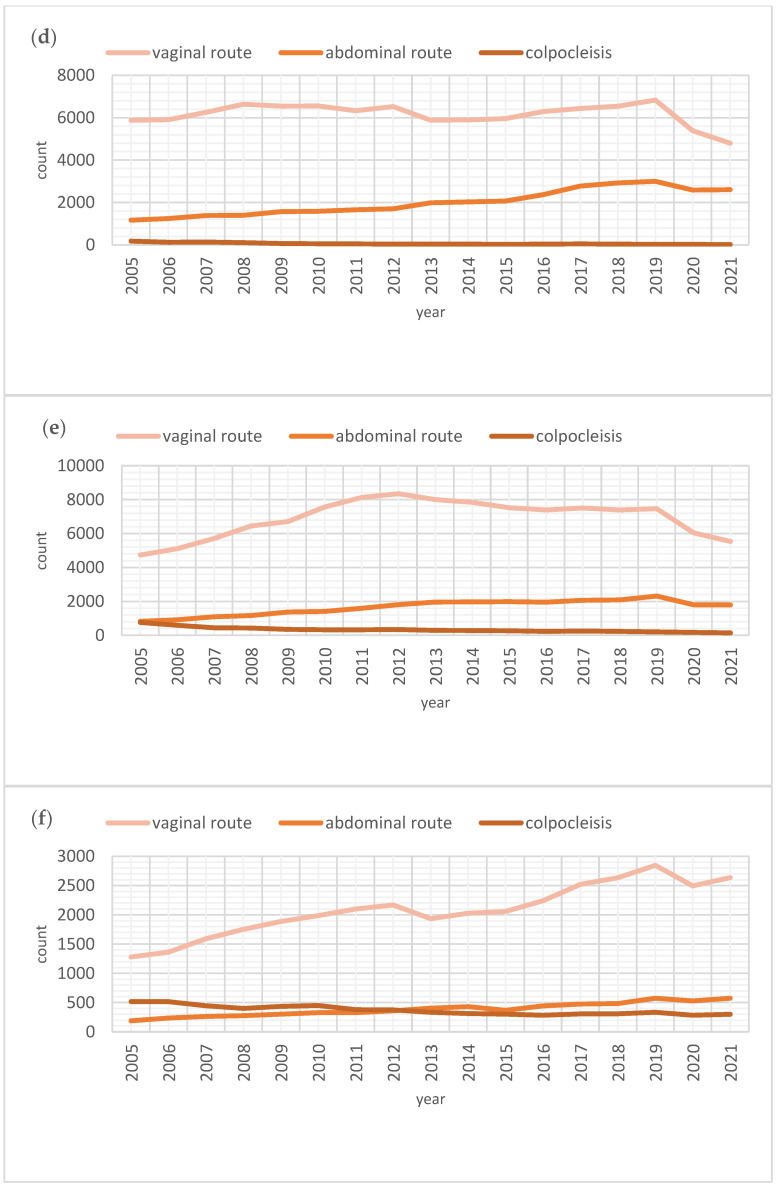
Surgical trends for apical POP repair. (**a**) Total number of apical POP repairs over the years. (**b**) Total number of apical POP repairs over the years among women under 50. (**c**) Total number of apical POP repairs over the years among women between 50 and 60. (**d**) Total number of apical POP repairs over the years among women between 60 and 70. (**e**) Total number of apical POP repairs over the years among women between 70 and 80. (**f**) Total number of apical POP repairs over the years among women over 80.

The total annual number of prolapse surgeries was 23,140 in 2005, demonstrating a general upward trend and reaching the peak of 36,173 procedures in 2019. However, a slight decrease was observed in subsequent years, with 29,617 and 28,617 surgeries performed in 2020 and 2021, respectively. Over the study period, obliterative surgery showed a significant decline, whereas the number of vaginal surgeries exhibited a fluctuating but lightly increasing pattern. In contrast, abdominal surgeries consistently increased, starting at 4070 annually in 2005 and peaking at 11,773 annually in 2019. Despite a minor decline in 2020 and 2021, with 9778 and 9859 procedures, the overall trend for abdominal surgeries showed a clear and substantial increase over the years (Figure 2).

**Figure 3:**
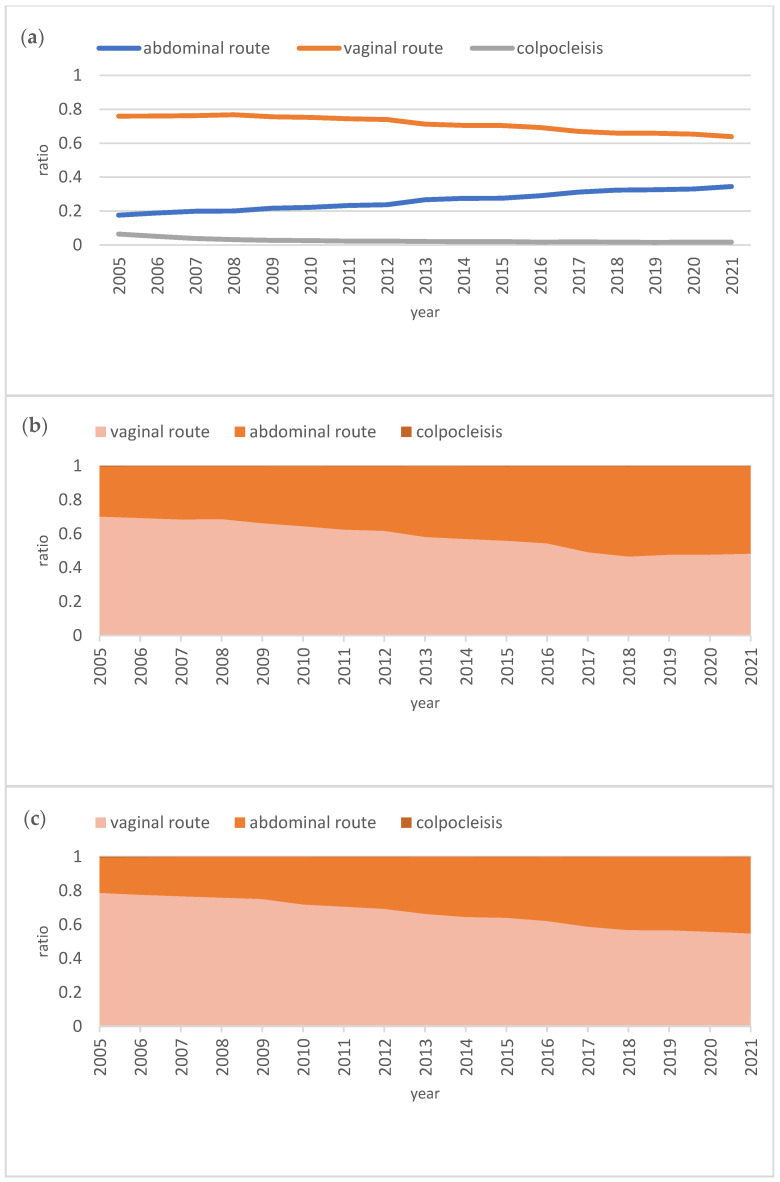

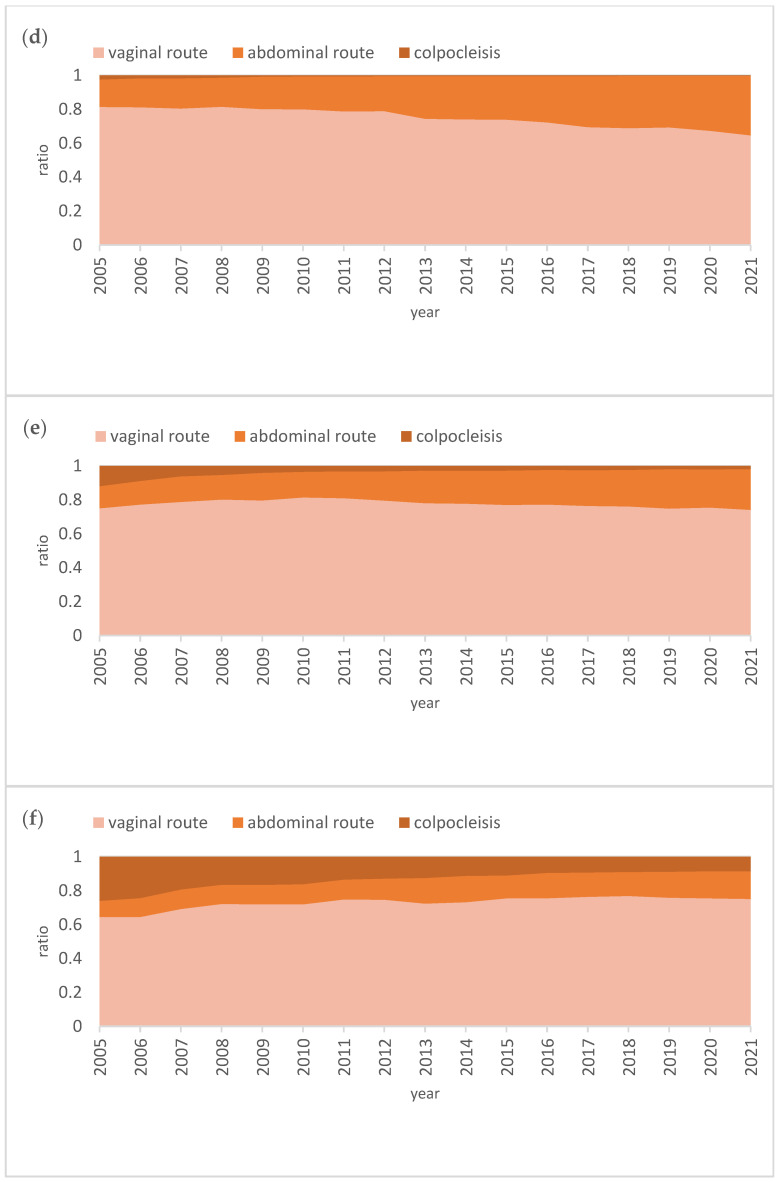
Surgical trends for apical POP repair. (**a**) Overall ratios of apical POP repairs over the years, including abdominal (β = 0.994, *p* < 0.001, R^2^ = 0.998) and vaginal (β = −0.982, *p* < 0.001, R^2^ = 0.965) routes. (**b**) Proportional distribution of apical POP repairs within the total over the years among women under 50. (**c**) Proportional distribution of apical POP repairs within the total over the years among women between 50 and 60. (**d**) Proportional distribution of apical POP repairs within the total over the years among women between 60 and 70. (**e**) Proportional distribution of apical POP repairs within the total over the years among women between 70 and 80. (**f**) Proportional distribution of apical POP repairs within the total over the years among women over 80.

Analyzing the ratios established to understand the relationship and proportional trends, it became evident that the proportion of abdominal surgeries relative to the total number of surgeries significantly increased over time (β = 0.994, *p* < 0.001, R^2^ = 0.998). In contrast, vaginal surgeries showed a tendency toward a gradual decrease in their proportion relative to the total over the same period (β = −0.982, *p* < 0.001, R^2^ = 0.965) (Figure 3).

The increasing trend of the abdominal route became particularly noticeable in the under-50 age group, where the proportion of abdominal surgeries reached parity with vaginal surgeries over the years, achieving 51% in 2021. This proportion similarly increased in the 50–60 age group, reaching 45% in 2021. In the 60–70 age group, while the trend of applying the abdominal route also showed growth, vaginal surgery remained the dominant approach. Similar trends were observed in the 70–80 and over 80 age groups (Figure 3).

The graphical representations of these trends highlight that obliterative surgery, specifically colpocleisis, became more prominent in older age groups over time. However, even in the over-80 age group, colpocleisis accounted for only 8.5%—its maximum value in 2021—compared to reconstructive surgery. This represented a significant decline from 26% in 2005 within the same age group (Figure 3).

## 4. Discussion

Pelvic organ prolapse has been a notable aspect of women’s health throughout history, even mentioned in some of the oldest recorded medical literature, such as the Egyptian papyri. Although the use of vaginal hysterectomy (VH) as a treatment for POP dates back to ancient times, the first vaginal hysterectomy for prolapse was performed during the early years of the Modern Era in 1861. And after that, the technique of vaginal hysterectomy was combined with anterior and posterior colporrhaphy in 1915 [20,21,22]. Meanwhile, attempts were made in 1832 to treat prolapse using an obliterative method, specifically by closing the labia majora. However, these methods were unsuccessful until the acceptable colpocleisis technique was established in 1876 [23]. With a greater understanding of pelvic anatomy, vaginal hysterectomy combined with colporrhaphy became the predominant operation for POP in the early 20th century. By the 1950s, vaginal apical fixation methods and abdominal fixation for prolapse were introduced [24,25].

By the beginning of the 21st century, the era of vaginal native tissue repair for POP in the surgical management of apical prolapse experienced two significant shifts: the introduction of vaginal mesh and the advent of advanced endoscopic surgery [26]. Data from the U.S. on this period underscore this trend, specifically illustrating a shift in vaginal surgery from vaginal hysterectomy to laparoscopic procedures and fixations, as demonstrated in a study covering the years 1976 to 2006 [17]. Boyles et al. similarly indicated in their study an increase in surgeries among older women, accompanied by a decrease in procedures for younger women, along with a shift towards laparoscopic surgery, while the rates for vaginal hysterectomy remained relatively stable [27]. Notably, both studies also demonstrate a significant decline in the trend of the “historical” obliterative vaginal surgery for POP from the late 20th century to the early 21st century.

The development of POP surgery has progressed rapidly over the past two decades, with changes in surgical practices and feasibility potentially evolving faster than anticipated. Synthetic materials began to be incorporated by surgeons as early as the late 20th century to improve the effectiveness of reconstructive surgery, given the reported high recurrence rates—reaching up to 50% in some cases [13,28]. The first meshes, designed as “kits” for prolapse treatment, entered the market in the early 2000s, with subsequent approval by the Food and Drug Administration (FDA) in 2002 [29].

The first Cochrane review published in 2004 addressing the surgical management of POP in women highlighted vaginal mesh surgery as achieving better success rates compared to vaginal native tissue repair while also emphasizing the limited evidence available and reporting the superiority of abdominal sacrocolpopexy in the treatment of vaginal vault prolapse over native tissue repair, whereby ‘Colpocleisis’ was mentioned in this review only once as an option [30].

Increasing complication rates, nonetheless, simultaneously accompanied the rapidly rising trend in vaginal mesh surgery. Consequently, the FDA issued consecutive warnings in 2008 and 2011, followed by a mesh ban in 2019 [14,31,32]. This ban was followed in other countries, such as the UK and Australia [33]. Moreover, the concept of ‘reconstructive surgery’, emphasizing the preservation of prolapsing organs such as the uterus and the avoidance of hysterectomy to reduce surgical morbidity, gained increasing popularity during this transitional period. Interest in uterus-sparing surgery continued to grow, particularly after numerous studies reported successful anatomical and functional outcomes following uterus-preserving POP repair in both young and older women [34]. Later studies also supported the rising trend of this approach, particularly from the patient’s perspective [35,36,37].

In our study, we analyzed a substantial amount of mandatory data to highlight changes in surgical practices during the last two decades in Germany, starting from 2005 to 2021, for treating apical POP, with a particular focus on obliterative surgery during the changing global mesh era. The rising trend in the abdominal approach continued even after the FDA warnings.

Data from countries primarily affected by the mesh ban highlight the usual changing trend toward abdominal surgery in the transition from the mesh to the non-mesh era, where notably, a reverse shift was also observed in the trend for ‘historical’ obliterative surgery, with increasing prevalence [15,16,38,39,40]. The U.S. data show a threefold increase in the rate of obliterative surgery from 2002 to 2012, primarily addressing its application in older women, though without a more specific age definition [16].

Our study also demonstrated a significant increase in the abdominal approach, particularly among younger women seeking treatment for POP. However, in contrast to other studies, we observed a notable decline in the use of colpocleisis. Based on our data, the option for colpocleisis appeared to be primarily considered for the age group over 80 despite the decreasing trend. Considering global data showing a growing preference for uterus-sparing POP surgery, our data indicated a halving of the Douglas reconstruction procedure over the study period. This can likely be attributed to the decreasing trend of vaginal hysterectomy for POP treatment, as Douglas reconstruction is most commonly a standard step in vaginal hysterectomy. Another study from a European country without a mesh ban, which examines the trend in apical prolapse treatment from 2010 to 2016, revealed that the number of colpocleisis cases slightly decreased while consistently maintaining very low case numbers throughout the study period. The study also highlighted a rising trend in the use of uterine-preserving options, specifically vaginal native tissue repair [41].

The U.S. trend following the mesh ban indicates a shift in the treatment of elderly patients, favoring vaginal surgery via native tissue repair (NTR) and the rising trend for abdominal repair via sacrocolpopexy, which was more commonly reserved for younger women during the period when the use of mesh significantly decreased, reflecting the evolving preferences for prolapse surgery among older women [42]. Our data indicated a significant preference for vaginal surgery among older women; however, the altered proportions that favor abdominal surgery may reveal different patterns in this group, suggesting a different safety perspective for abdominal surgery in treating apical prolapse in the elderly population in Germany.

The growing preference for obliterative surgery among some surgeons and in certain countries following the mesh ban may be linked to the decreasing number of treatment options for such patients, as data from the mesh era indicated that the preference for mesh surgery was mainly among women over 65 years old [43]. The declining trend in obliterative surgery in Germany, in contrast to other data showing an increasing trend, can certainly be explained by the continued use of transvaginal mesh surgery with the goal of uterine preservation, which is appropriately indicated even for the older population, including those over 80 years old. This trend was demonstrated in another study by our study group, which showed the trend for mesh surgery in Germany [13].

The Cochrane Database regarding apical prolapse treatment also showed the shift in scientific evidence over time, addressing transvaginal mesh surgery and emphasizing that the limited evidence did not support the use of transvaginal mesh compared to native tissue repair for apical vaginal prolapse anymore. Additionally, it stated that sacrocolpopexy was associated with a lower risk of prolapse awareness, recurrent prolapse on examination, repeat surgery for prolapse, and postoperative stress urinary incontinence (SUI) compared to a variety of vaginal interventions [44,45]. The German preferences seemed to have developed in line with guidelines, with more than 50% of surgical approaches for apical prolapse treatment performed abdominally on women under 50.

An additional factor that might help explain the differing trends in obliterative surgery could be the perspective on sexuality, which plays a key role in the treatment of prolapse and remains a poorly understood aspect of the aging process. The WHO defines sexuality as a central aspect of human life, encompassing diverse elements such as sexual orientation, eroticism, and intimacy [46]. The demographics have changed significantly over the past century; however, there remains a common misconception that individuals become asexual as they age. As described by Rosanne Freak-Poli, sexual activity is associated with partner availability and better health, rather than age, countering stereotypes of decline in sexual behavior and normalizing sexual activity and desire in later life [19]. As the population ages, the ratio of men to women will also shift from 76 to 100 in 2013 for those aged 60 and older to 87 to 100 by 2050. This change is due to increased life expectancy and better healthcare [8]. Contrary to common misconceptions, sexual activity does not necessarily diminish with age, and many older individuals remain sexually active. As highlighted in the first Cochrane review, the ‘restoration or maintenance of normal sexual function’ is considered one of the key aims of surgery in the management of POP [30]. From this perspective, maintaining sexuality can be made a key goal in the treatment of POP. However, colpocleisis presents a significant drawback in this regard, as it results in the impossibility of sexual intercourse following the procedure. The higher regret rates following colpocleisis, which reached up to 50% as measured by the PFDI scale, were observed in the long-term follow-up study by Winkelman et al.; the loss of sexuality, on the other hand, was not a reason for regret after colpocleisis in this study group [47]. The higher regret rates over the loss of coital ability are reported in the study by Pechmann et al., with approximately 12% of patients experiencing this regret. However, half of these patients indicated they would undergo the colpocleisis procedure again [48]. A further study reporting the outcomes of 58 women who underwent colpocleisis showed no regret among the participants, with all patients stating they would choose to undergo the colpocleisis procedure again [49]. In this regard, among other findings, another study in the literature on POP in elderly patients emphasized the sexual consequences associated with obliterative surgery, stating the following: ‘Of course, this is a procedure that every urogynecology surgeon should be able to perform, but it should not be offered regularly to patients’ [50]. From this perspective, the loss of sexuality did not seem to play a decisive role in an appropriate patient group; however, based on our study data, it may still play a role in the preferences of the treating physician in Germany.

A further factor that might explain the shift between obliterative and reconstructive surgery is the complication rate in older women. Data demonstrate that obliterative surgery in older women was linked to a lower incidence of high-grade complications compared to reconstructive surgery [51]. Some data, however, indicated that frailty, rather than age or the type of surgical procedure, was more strongly associated with postoperative complications [52]. In this regard, however, the reasons underlying the increase in the prevalence of colpocleisis in certain countries were not readily ascertainable from the available published data. It is important to acknowledge at this point that our study’s limitation is the lack of information on operation indications and data regarding comorbidities and surgery complications, which limits the depth of this analysis in this context. However, the increasing number of publications on obliterative surgery indicates that interest in this method has risen significantly over the past two decades globally [53].

Furthermore, concerning the global trend, the *Trends in Pelvic Organ Prolapse Management in Latin America* study showed crucial results, which may help clarify the impact of surgical training and expectations on surgical trends. Based on the study data, every second surgeon would offer an obliterative option, while only 1 in 5 participants would prefer it for future training in POP repair. Two-thirds of the participants in this study, particularly those with proficiency in urogynecology, were younger than 50 years old [54]. Additional comparative data showed that older surgeons performed colpocleisis more frequently than younger surgeons, reflecting the different types of procedures offered as treatment options [55]. Published literature also support the idea that surgical volume is critical for achieving and maintaining proficiency. The low volume of surgeries performed by specialists could potentially impact the proficiency of trainees nowadays and in the future [56]. U.S. data show that the trend in resident surgical training in urogynecology is declining [57,58]. Similarly, a German study highlighted the gap between demand and reality in urogynecological training in Germany, where despite higher interest in urogynecology, there was a lack of standardized training in the respective departments [59]. The surgical practice and training will undoubtedly influence the changing future trends in urogynecologic surgery.

Our study examined and discussed the shifting trends in prolapse surgery from a historical perspective, highlighting various approaches to treating apical POP. By analyzing a comprehensive dataset over several years, particularly concerning the evolving mesh era, we recognized this as one of the key strengths of our study. Our findings indicate that the surgical approach to treating apical prolapse in Germany has evolved in accordance with global developments over the years, marking a period of significant changes during the mesh era. Although the absolute numbers may still be relatively small in some countries, the proportionate rise in obliterative vaginal surgery is significant. Possible explanations for this trend include the relative and absolute decrease in using mesh kits for POP surgery in these countries following FDA warnings, the mesh ban, and the aging population. Additionally, the increasing demand for surgery may be attributed to improved awareness among the elderly, in contrast to previous generations. Our data similarly showed a rising number of prolapse surgeries, which is highly compatible with an aging German population.

## 5. Conclusions

Pelvic floor disorders, including POP, are a significant public health concern that adversely affects the quality of life for millions of adult women. The anticipated rise in the number of women experiencing PFDs in the coming decades will increase the demand for healthcare providers trained in urogynecology. However, as the approach to prolapse surgery has evolved over time, driven by evidence-based findings, specialists should be prepared to perform both reconstructive and obliterative surgeries. It is crucial to structure training programs effectively to ensure that future pelvic surgeons receive proper education.

## Figures and Tables

**Table 1:** Main OPS codes for pelvic organ prolapse (POP) surgeries and their corresponding procedures.

OPS-Codes		Procedures
5-703 5-703.0	obliterative	Vaginal closure and (sub-)total colpectomy Colpocleisis
5-704.4	reconstructive (vaginal/abdominal)	Vaginal stump fixation
5-704.5	reconstructive (vaginal/abdominal)	Cervical stump fixation
5-704.6	reconstructive (vaginal/abdominal)	Uterus fixation
5-707.1	reconstructive (vaginal)	Douglas reconstruction
5-707.2	reconstructive (vaginal/abdominal)	Enterocele repair without alloplastic material
5-707.3	reconstructive (vaginal/abdominal)	Enterocele repair with alloplastic material

## Data Availability

All data are included in the study.
